# OTEH-7. Molecular characterization of tumor stiffness in glioblastoma

**DOI:** 10.1093/noajnl/vdab070.046

**Published:** 2021-07-05

**Authors:** Skarphedinn Halldorsson, Siri Fløgstad Svensson, Henriette Engen Berg, Denise Wolrab, Frode Rise, Alistair Wilkins, Steven Ray Wilson, Michal Holcapek, Kyrre Eeg Emblem, Einar O Vik-Mo

**Affiliations:** 1 Institute for Surgical Research, Vilhelm Magnus Laboratory, Oslo University Hospital, Oslo, Norway; 2 Department of Diagnostic Physics, Clinic for Radiology and Nuclear Medicine, Oslo University Hospital, Oslo, Norway; 3 Department of Chemistry, Section for Chemical Life Sciences, University of Oslo, Oslo, Norway; 4 Faculty of Chemical Technology, Department of Analytical Chemistry, University of Pardubice, Pardubice, Czech Republic; 5 Department of Chemistry, University of Oslo, Oslo, Norway; 6 School of Science and Engineering, University of Waikato, Hamilton, New Zealand

## Abstract

Tumor heterogeneity is one of the hallmarks of glioblastoma multiforme (GBM). Morphology within a given GBM tumor can be extremely variable where some regions of the tumor have a soft, gel-like structure while other areas are dense and fibrous. Abnormal mechanical stress and tissue stiffening caused by cancer proliferation are believed to affect vascularity by compressing structurally weak blood vessels and restricting the supply of nutrients and oxygen to the tissue. These effects contribute to a hypoxic microenvironment that promotes disease progression and chemoresistance. The genetic and molecular mechanisms that govern tissue stiffness within GBM tumors, however, are largely unknown. Magnetic Resonance Elastography (MRE) is an emerging technique for quantifying tissue stiffness non-invasively.

We have evaluated 10 GBM patients by MRE imaging obtained prior to surgical resection. During surgery, 2–7 stereotactically navigated biopsies were collected from locations within the tumor with varying degrees of measured stiffness. Biopsies were processed to extract RNA, proteins, polar metabolites and lipids. Biomolecules were analyzed on relevant -omics platforms (RNA sequencing, MS-proteomics and lipidomics, NMR of polar metabolites).

Differential expression and gene set enrichment analysis of patient paired biopsies indicate an overall increase in macrophage infiltration and extracellular matrix re-organization associated with increased tumor stiffness. Among the most highly upregulated genes in stiff tumor tissue were lymphatic endothelial hyaluronic acid receptor 1 (LYVE-1) and macrophage receptor with collagenous structure (MARCO), both of which have been associated with immune cell infiltration and tissue stiffness.

Our preliminary findings offer novel insights into tumor morphology in GBM that can be inferred from imaging prior to surgery. This can be used to identify tumor regions with high risk of progression and infiltration, thereby informing and guiding surgical strategy and may ultimately lead to novel treatment strategies.

